# CircMUC16 promotes autophagy of epithelial ovarian cancer via interaction with ATG13 and miR-199a

**DOI:** 10.1186/s12943-020-01163-z

**Published:** 2020-02-28

**Authors:** Xiaoling Gan, Hongtao Zhu, Xingwei Jiang, Samuel C. Obiegbusi, Min Yong, Xingtao Long, Jianguo Hu

**Affiliations:** 1 Department of Obstetrics and Gynecology, The Second Affiliated Hospital, Chongqing Medical University, Chongqing, China; 2 Department of Gynecologic Oncology, Chongqing University Cancer Hospital and Chongqing Cancer Institute and Chongqing Cancer Hospital, Chongqing, 400030 People’s Republic of China

**Keywords:** CircMUC16, Autophagy, Beclin1, RUNX1, miR-199a-5p

## Abstract

**Background:**

Circular RNA (circRNA) has been proven to play a significant role in multiple types of cancer. However, the expression and role of circRNAs in epithelial ovarian cancer (EOC) remains elusive.

**Methods:**

CircRNA and mRNA expression profiles of EOC were screened with sequencing analysis. Gene silencing and over-expression were used to study circRNA function. Cell proliferation and Matrigel invasion assays were used to detect cell proliferation and invasion, respectively. The expression of circRNAs, mRNAs and miRNAs was detected using qPCR. The location of circRNAs was detected using FISH. The expression of proteins was detected using western blot and immunohistochemistry.

**Results:**

CircMUC16 had increased expression in EOC tissues as compared to healthy ovarian tissues. The expression of circMUC16 was linked to the progression in stage and grade of EOC. Hence, silencing circMUC16 suppressed autophagy flux of SKOV3 cells. In contrast, ectopic expression of circMUC16 promoted autophagy flux of A2780 cells. CircMUC16-mediated autophagy exacerbated EOC invasion and metastasis. Mechanistically, circMUC16 could directly bind to miR-199a-5p and relieve suppression of target Beclin1 and RUNX1. In turn, RUNX1 elevated the expression of circMUC16 via promotion of its transcription. CircMUC16 could directly bind to ATG13 and promote its expression.

**Conclusion:**

This study demonstrated that circMUC16 regulated Beclin1 and RUNX1 by sponging miR-199a-5p. The data suggested that circMUC16 could be a potential target for EOC diagnosis and therapy.

## Background

Ovarian carcinoma is one of the most common gynecological malignant tumors with a high mortality rate [1]. Despite rapid progress in the chemotherapy and radiotherapy of ovarian carcinoma, patients with recurrent ovarian cancer are essentially incurable [2]. Unfortunately, the molecular etiology of ovarian cancer remains elusive [3].

Circular RNA (circRNA) is a class of noncoding RNAs discovered in recent years [4]. CircRNAs have various biological functions, which includes regulation of cell proliferation, invasion, apoptosis, etc. [5]. CircRNA ITCH inhibited the malignant behavior of esophageal squamous cell carcinoma through suppression of Wnt/β-catenin pathway [6]. CircHIPK3 regulates the growth of cells by binding directly to miR-124 and inhibits its activity [7]. CircRNA_001059 and circRNA_000167 are involved in the development of radiation resistance [8]. CircRNA ITCH inhibits proliferation and promotes apoptosis of human epithelial ovarian cancer (EOC) cells by sponging miR-10a [9]. The expression of circLARP4 decreases in ovarian cancer, and circLARP4 serves as a potential biomarker of ovarian cancer prognosis [10]. CircITCH is related to tumor size, FIGO stage and overall survival. It also suppresses cell proliferation and promotes apoptosis in EOC [11].

However, there is no complete and systematic study on the expression and role of circRNAs in ovarian cancer. In this study, we performed genome-wide circRNA analysis of EOC tissues and healthy ovarian tissues via next-generation sequencing (NGS) technology. The aim was to investigate the role and demonstrate the potential mechanism of circRNAs in EOC.

## Materials and methods

### Ovarian tissues

The ovarian and serum samples were obtained from the Department of Obstetrics and Gynecology, Second Affiliated Hospital, Chongqing Medical University. The Ethics Committee of Chongqing Medical University approved this study. Three pathologists confirmed the pathological diagnosis of ovarian cancer.

### RNA sequencing analysis

First, we characterized circRNA transcripts via sequencing analysis of ribosomal RNA and linear RNA. Total RNA was extracted from three EOC tissues and four healthy ovarian tissues. Each sample was sequenced on Illumina HiSeq and yielded an average of 57.74 million reads, which were mapped to the human reference genome (GRCh38/hg38) by TopHat2. Qualified reads were sent to CIRCexplorer v2.2.3 pipeline to identify and quantify circRNAs with default parameters. A circRNA was called with the support of a minimum of two unique back-spliced reads. The whole process of library construction, sequencing and data analysis was performed at Shanghai Lifegenes Technology Co., Ltd.

### Cell culture, transfection procedure, and reagents

SKOV3 human ovarian cancer cell line derived from the ascites from a 64 year old caucasian female with an ovarian tumor. The ES-2 cell line was established from a surgical tumor specimen taken from a 47 year old black woman. The A2780 cell line was established from tumor tissue from an untreated patient. CAOV-3 human ovarian cancer cell line derived from a 54 years old caucasian female with ovarian adenocarcinoma. Human ovarian cancer cells were cultured in RPMI 1640 medium (Sigma-Aldrich, R8758), with 10% fetal bovine serum and streptomycin. The cells were incubated under 5% CO2 at 37 °C [12]. Lentiviral shRNA interference vectors targeting circMUC16 (named LV2–1 and LV2–2) and the circMUC16-lentiviral expression vector (named LV6) were purchased from Genepharma. MIR199A mimics and inhibitors were synthesized by Genepharma. We constructed pCMV5-Beclin1 for overexpression of Beclin1. The siRNA was synthesized by Genepharma (Shanghai, China). The sequences are as follows: CircMUC16–1: 5′-TGACCTTGCTCAGGCCCGA-3′; CircMUC16–1: 5′-ACCTTGCTCAGGCCCGAGA-3′; RUNX1: 5′-GGAUUUCUGUUGUGUUUAAAU-3′; BECLIN1: 5′-GGUGUUUGAUACUGUUUGAGA-3′; ATG5: 5′-CAAUCCCAUCCAGAGUUGCUUGUGA-3′; and NC (negative control) siRNA: 5′-UUCUUCGAAGGUGUCACGUTT-3′.

### Tandem mRFP-GFP-LC3 fluorescence

We used a tandem mRFP-GFP-tagged LC3 to monitor autophagy flux based on our previous study [12]. SKOV3 cells were infected with mRFP-GFP-LC3 and LV2–1, LV2–2 or LV2-NC. A2780 cells were infected with mRFP-GFP-LC3 and LV6 or LV6-NC. mRFP-GFP-LC3 distribution in cells was analyzed by confocal microscope. Image pro plus 6.0 software was used to quantify the LC3 dots. All experiments were repeated three times [12].

### RIP assay

Biotin-labelled circMUC16 probe was purchased from Genepharma. The specific operation protocol was in accordance with a previous study [13]. Finally, the expression of miRNA was detected using qPCR.

### CircRNA pulldown

The specific steps of circRNA pulldown are described in our previous study [14]. Briefly, the specific proteins were identified using SDS-PAGE gel electrophoresis, silver stain and MALDI-TOF-MS [14].

### qPCR

Total RNA was isolated using a high-purity total RNA rapid extraction kit (Bioteke Corporation, RP1201) following the manufacturer’s instructions. RNA was reverse-transcribed into cDNA using the iSCRIPT cDNA synthesis kit (Bio-Rad Laboratories, 4,106,228). The forward primer for circMUC16 sequence was 5′-CTGCTCAGGCCTGTGTTC-3′, and the reverse primer sequence was 5′-GGGGCCCAGCTCTTCA-3′. The real-time PCR was performed using the All-in-One qPCR mix kit. Each sample was analyzed in triplicate. The primers were purchased from Genepharma. The 2^-ΔΔCT^ method was used to quantify the gene expression [14].

### Chromatin immunoprecipitation (ChIP) assay

We used ChIP assay kit (Cell Signaling Technology, 9003) to conduct a ChIP assay according to the manufacturer’s instructions. Briefly, cross-linked chromatin was sonicated into 200–1000-bp fragments. Then, the chromatin was immunoprecipitated using anti-RUNX1 antibody (ab23980, Abcam). qPCR was performed as described earlier [12].

### Dual-luciferase reporter gene assay

Luciferase reporter gene assay was performed using the dual-luciferase reporter assay system (Promega Corporation, E1910) according to the manufacturer’s instructions. The wild-type or mutant reporter of circMUC16, Beclin1 and RUNX1 was constructed. Then, the reporter was co-transfected into SKOV3 cells in 24-well plates with 100 nM miR-199a or 100 nM miR-NC and Renilla plasmid using Endofectin-Plus (Gene-Copeia, Z01010A). All experiments were repeated three times [12].

### Western blotting

The expressions of Beclin1, RUNX1, ATG14, and GAPDH proteins were detected by western blot. The primary antibodies used included rabbit monoclonal anti-Beclin1 (Abcam, ab210498); rabbit polyclonal RUNX1 (Proteintech, 25,315–1-AP); rabbit polyclonal ATG14 (Abcam, ab227849), and rabbit monoclonal anti-GAPDH (Abcam, ab181602). The band density was analyzed using a gel imaging system and compared with the internal control.

### EdU assay

The cell proliferation was detected using Cell-Light Apollo567 in vitro kit (RiboBio, Guangzhou, China). Ovarian cancer cells were cultured in 1640 medium. EdU was applied at 20 μM. The cells were fixed with 4% paraformaldehyde and stained with Apollo 567 and Hoechst 33342 [12].

### Matrigel invasion assays

Matrigel invasion assays were used to determine the cellular invasion ability as described in our previous study [12]. A total of 5 × 104 cells were seeded into the top chamber of the transwell filter and incubated. After 48 h, the cells on the lower side of the chamber were fixed with 4% paraformaldehyde, stained with 0.5% crystal violet (Beyotime Institute of Biotechnology, C0121), and counted using a microscope [12].

### Fluorescence in situ hybridization (FISH) assay

Cy3-labelled probe against circMUC16 (5’CY3-TCTTTCTCGGGCCTGAGCAAGGTCAGT- 3’CY3) and FITC-labeled probe against miR-199a were obtained from Genepharma. The FISH assay was performed following the manufacturer’s guide and instructions (F03401, Genepharma). Cell nuclei were stained with Hoechst 33342. Finally, images were obtained on a confocal microscope [15, 16]. All experiments were repeated three times.

### Mouse xenograft model

The xenograft model was established as described in our previous study [12]. All animal experimental procedures were conducted with the approval of the Committee on the Use and Care of Animals (Chongqing Medical University, Chongqing, China), based on the institution’s guidelines. SKOV3 cells were infected with LV2-NC and LV2–1, and intraperitoneally injected into six-week-old BALB/c nude mice (5 × 106 cells). After five weeks, the animals were examined. Then, the number of mice with ascites were counted and weighed [12].

### Statistical analysis

All statistical analyses were performed with SPSS version 17.0 software (Chicago, IL). Statistical analysis was conducted by Student’s t-test or analysis of variance (ANOVA). The chi-square test was used to compare the associations between circMUC16 expression and the clinicopathological variables of ovarian cancer samples. Data were presented as mean ± standard deviation. A two-sided *p* < 0.05 was considered significant.

## Results

### Identification of differentially expressed circRNAs in epithelial ovarian cancer tissues

**Fig. 1:**
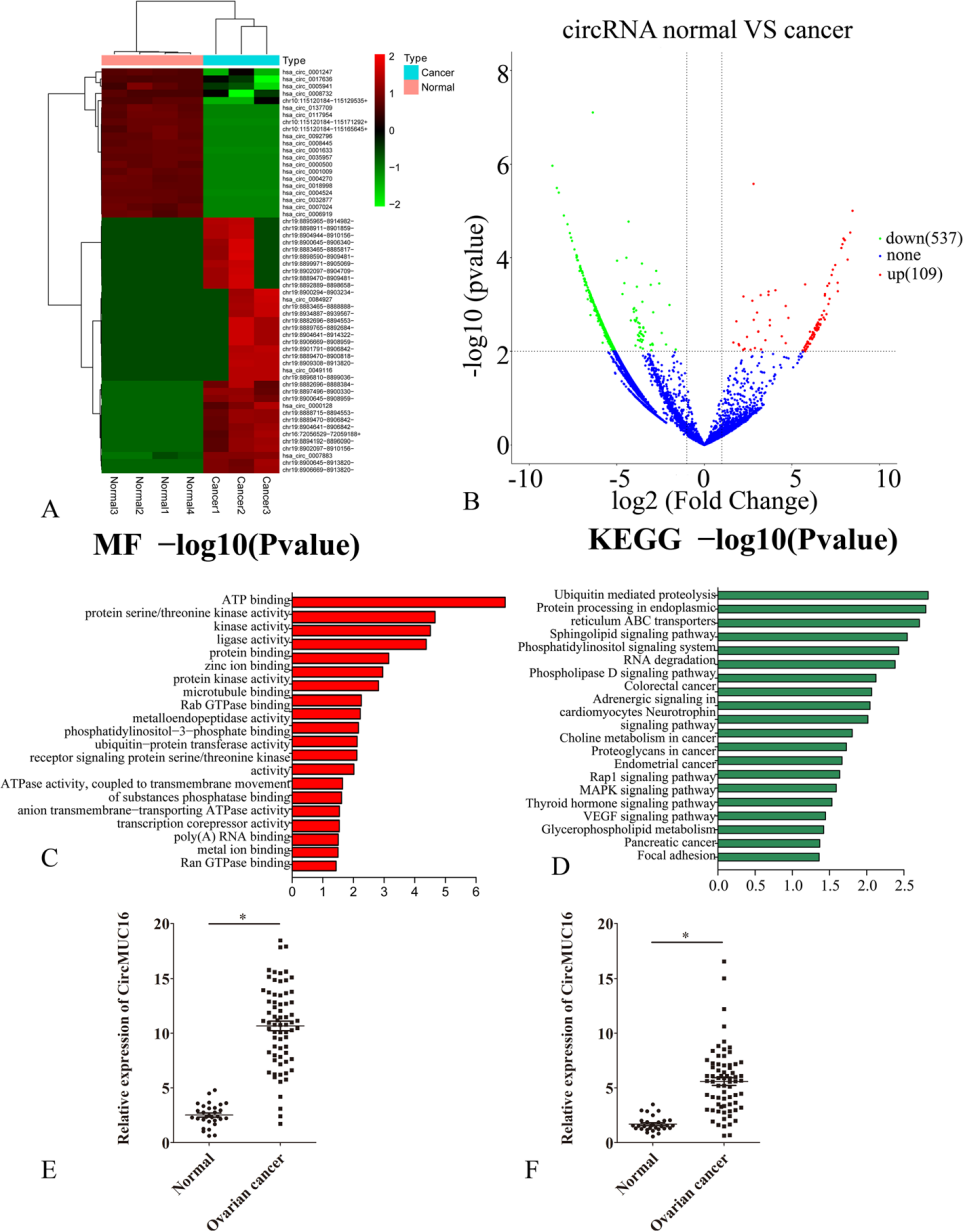
**a** Hierarchical clustering of circRNA differential expression profiles between three EOC samples and four normal ovarian samples. The heat maps are generated from differentially expressed circRNAs (top 50). Red and green colors indicate relative expression above or below the average. **b** Volcano map of deferentially expressed circRNAs (top 50). **c** Functional analysis of differentially expressed circRNAs (top 50). **d** The top 50 expressed circRNAs were analyzed by KEGG pathway. (D) CircMUC16 expression was detected in 30 normal ovarian tissues and 70 EOC samples. **e** The expression of circMUC16 in 30 paired serum samples with normal ovarian tissues and 70 serum samples with epithelial ovarian cancer tissues. Error bars represent the standard error. The symbols * and ** show *p* < 0.05 and 0.01, respectively

A total of 9151 distinct circRNA candidates were found in all the samples. Of these, 110 circRNAs were elevated, and 538 circRNAs were reduced; 2840 mRNAs were elevated, 4705 were reduced; 441 LncRNAs were elevated, and 467 were down-regulated (Fig. 1a). The variation of circRNA expression between EOC and healthy ovarian tissues was assessed using the Scatter plot (Fig. 1b). GO analysis revealed that these circRNAs were involved in many cellular processes including ATP binding, protein serine/threonine kinase activity, protein binding, zinc ion binding, protein kinase activity, etc. (Fig. 1c). Pathway analysis revealed that the those circRNAs were related to the VEGF pathway, ubiquitin-mediated proteolysis, Rap1 signaling pathway, platinum drug resistance, MAPK signaling pathway, etc. (Fig. 1d).

The expression of circRNAs generated by MUC16 was significantly elevated in EOC tissues as compared to ovarian tissues. Hsa_circ_0049116 is also derived from MUC16, and its expression was increased in EOC tissues. The information of hsa_circ_0049116 can be queried in both circbank and circbase. Therefore, we aimed to study the expression pattern, function and role of hsa_circ_0049116 (which we named circMUC16). We first verified the expression of circMUC16 in EOC samples. The expression of circMUC16 was significantly elevated in EOC samples than in healthy ovarian tissues (Fig. 1e). The expression of circMUC16 was also increased in serum of patients with EOC than in serum derived from healthy population (Fig. 1f). The ovarian cancer tissues were divided into two groups to investigate the correlation between the expression of circMUC16 with pathological features of high circMUC16 group and low circMUC16 group. High level of circMUC16 was positively correlated with tumor stage (stage I/II versus III/IV) and tumor grade (grades 2–3 versus 1; Table 1).

**Table 1:** Association of CircMUC16 expression with clinicopathological characteristics in 70 patients of EOC

	No. of patients	CircMUC16 expression		*P* value
	(*n* = 100)	Low no.(%)	High no.(%)	
Characteristics				
Age (years)				> 0.05
< 50	48	23(47.92%)	25(52.08%)	
≥ 50	52	24(46.15%)	28(53.85%)	
Normal ovarian	30	28(93.33%)	2(6.67%)	< 0.05
Cancer tissues	70	19(27.14%)	51(72.86%)	
FIGO stage				
I/II	40	15(37.50%)	25(62.50%)	< 0.05
III/IV	30	4(13.33%)	26(86.67%)	
Grade				
1	16	12(75.00%)	4(25.00%)	
2	21	5(23.81%)	16(76.19%)	
3	33	2(6.06%)	31(93.94%)	
		Grade 2–3 versus 1		< 0.05

### CircMUC16 mediated autophagy flux of epithelial ovarian cancer

**Fig. 2:**
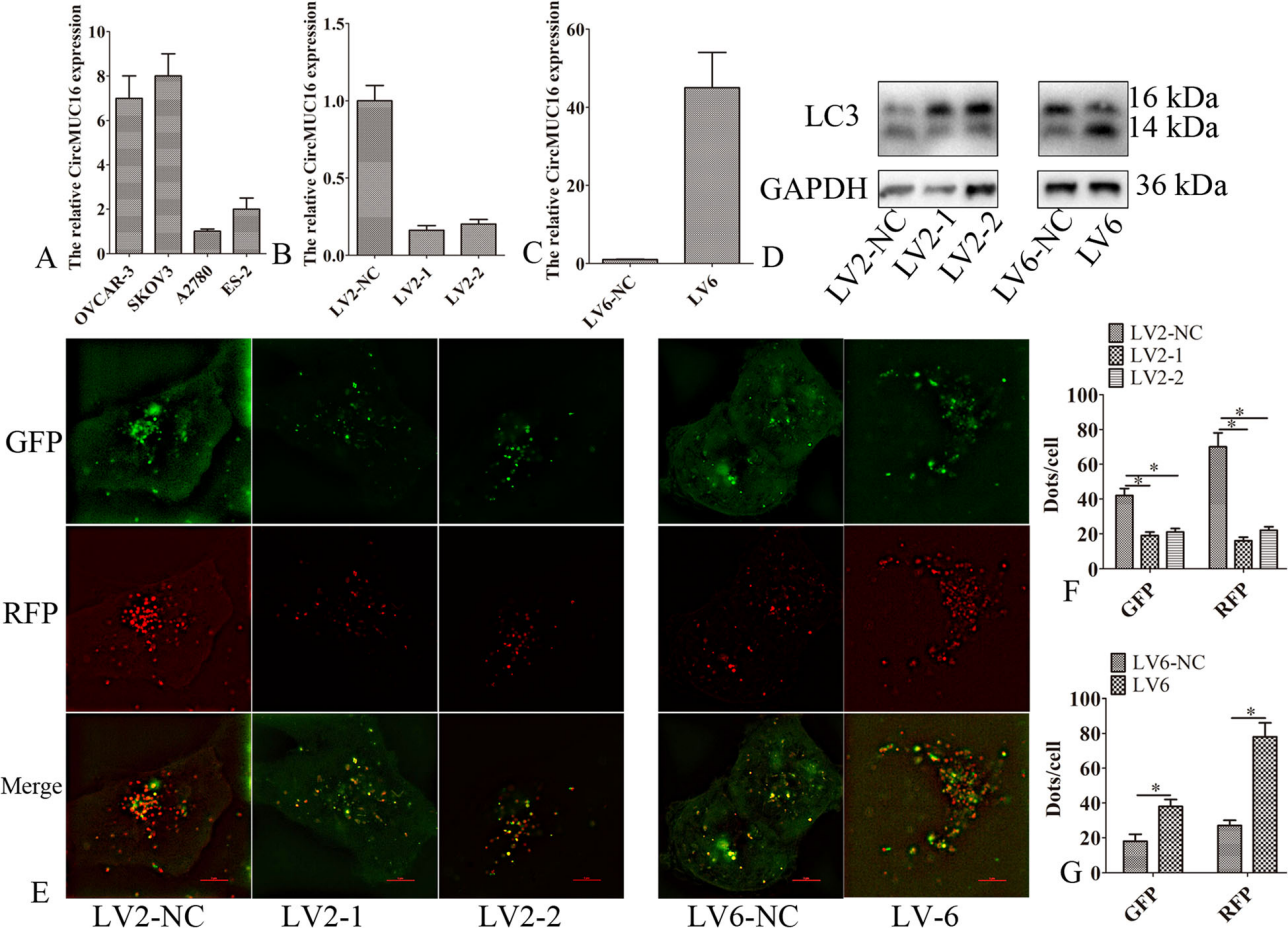
CircMUC16-mediated autophagy flux of epithelial ovarian cancer. **a** CircMUC16 expression was determined in four cell lines by qPCR. **b** CircMUC16 expression was detected in SKOV3 cells. **c** The expression of circMUC16 was detected in A2780 cells. **d** Western blot was used to detect the expression of LC3-II after silencing or ectopic expression of circMUC16. **e** mRFP-GFP-LC3 distributions in SKOV3 and A2780 cells were analyzed by confocal microscopy after silencing or ectopic expression of circMUC16. The symbols * and ** show *p* < 0.05 and 0.01, respectively. Scale bar: 5 μm

We first detected the expression pattern of circMUC16 for determine the role of circMUC16 in EOC. The expression of circMUC16 in SKOV3 and OVCAR-3 cells was higher than in A2780 and ES-2 cell lines (Fig. 2a). The expression of circMUC16 was reduced in SKOV3 cells after silencing circMUC16 (Fig. 2b). The expression of circMUC16 was elevated in A2780 cells after ectopic expression of circMUC16 (Fig. 2c). The apoptotic rate did not change in SKOV3 cells after silencing circMUC16. Also, apoptotic rate did not change in A2780 cells after ectopic expression of circMUC16 (data not shown). So, we asked whether circMUC16 mediated autophagy. The expression of LC3-II decreased in SKOV3 cells after silencing circMUC16. The expression of LC3-II was increased in A2780 cells after ectopic expression of circMUC16 (Fig. 2d).

**Fig. 3:**
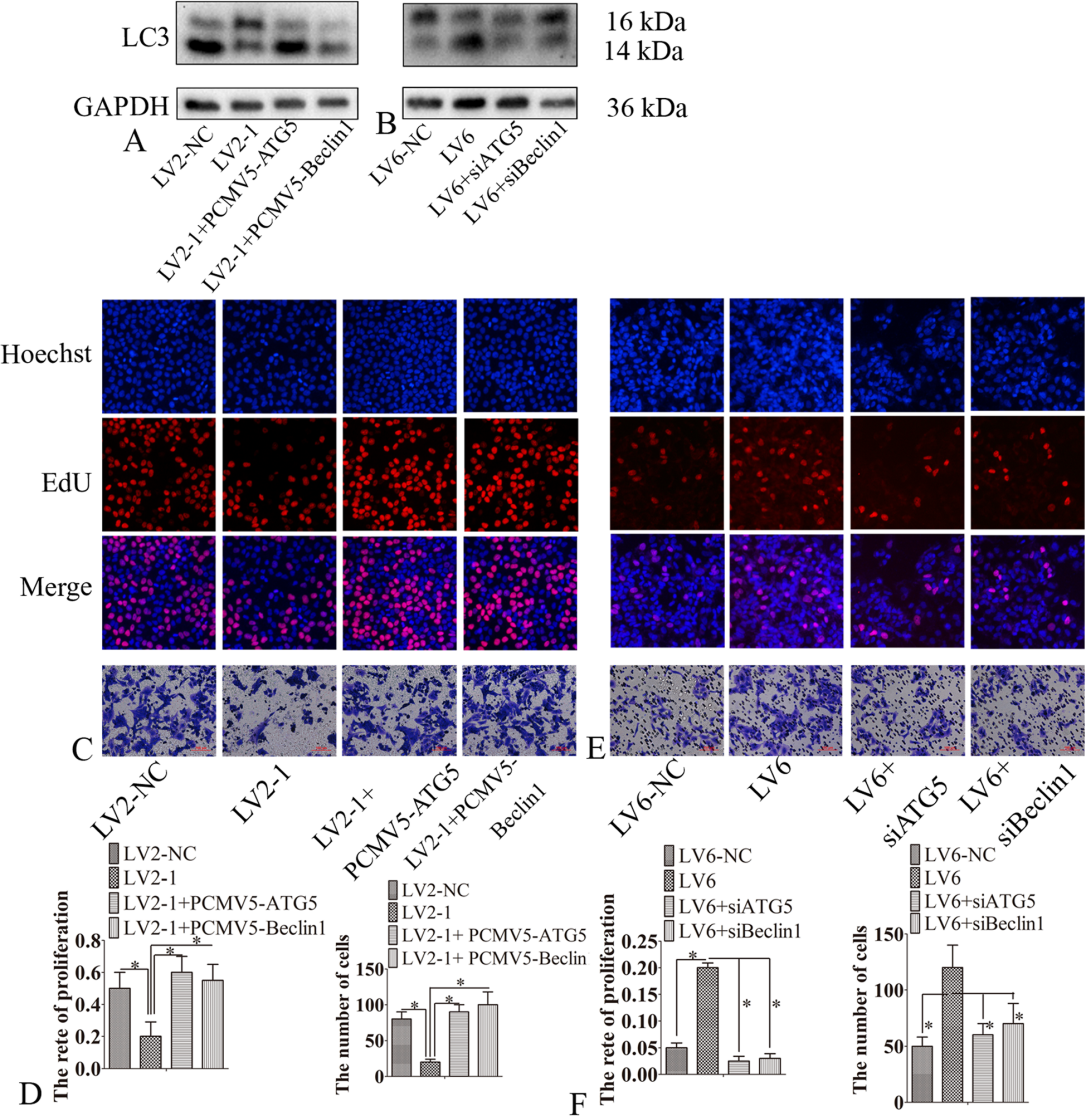
CircMUC16-mediated autophagy promoted cellar proliferation and invasion. **a**, **b** SKOV3 cells were transfected with LV2-NC, LV2–1, LV2–1 + PCMV5-ATG5, or LV2–1 + PCMV5-Beclin1. A2780 cells were transfected with LV6-NC, LV6, LV6 + ATG5 siRNA, or LV6 + Beclin1 siRNA. Then, western blot was used to detected the expression of LC3. **c**-**f** SKOV3 cells were transfected with LV2-NC, LV2–1, LV2–1 + PCMV5-ATG5, or LV2–1 + PCMV5-Beclin1. A2780 cells were transfected with LV6-NC, LV6, LV6 + ATG5 siRNA, or LV6 + Beclin1 siRNA. Then, the cellular proliferation and invasion was detected. Error bars represent the standard error. The symbols * and ** indicate *p* < 0.05 and 0.01, respectively. Scale bar: 100 μm

Autophagy activation or inhibition of autophagy flux lead to LC3-II accumulation. So, we monitor autophagy flux using the mRFP-GFP-LC3 reporter. The red puncta were decreased in SKOV3 cells after knockdown of circMUC16. However, the red puncta were increased in A2780 cells after ectopic expression of circMUC16. These results showed that circMUC16 activated autophagy (Fig. 3e-g).

### CircMUC16-mediated autophagy accelerated proliferation and invasion of epithelial ovarian cancer cells

Since circMUC16 was found to regulate autophagy, we investigated whether circMUC16-mediated autophagy was involved in cellular proliferation and migration. The expression of LC3-II was decreased in SKOV3 cells after knockdown of circMUC16. However, ectopic expression of ATG5 or Beclin1 partly reversed this effect (Fig. 3a). The LC3-II expression was elevated in A2780 cells infected with LV6-circMUC16. Consequently, when ATG5 or Beclin1 was silenced, this effect was partly reversed (Fig. 3b). The cellular proliferation and invasion of A2780 cells infected with LV6 was increased. Also, when autophagy was inhibited via silencing ATG5 or Beclin1, this effect was partly retarded (Fig. 3c-f). Hence, circMUC16-mediated autophagy promoted proliferation and invasion of ovarian cancer.

### CircMUC16-miR-199a-5p-Beclin1 axis regulated autophagy

**Fig. 4:**
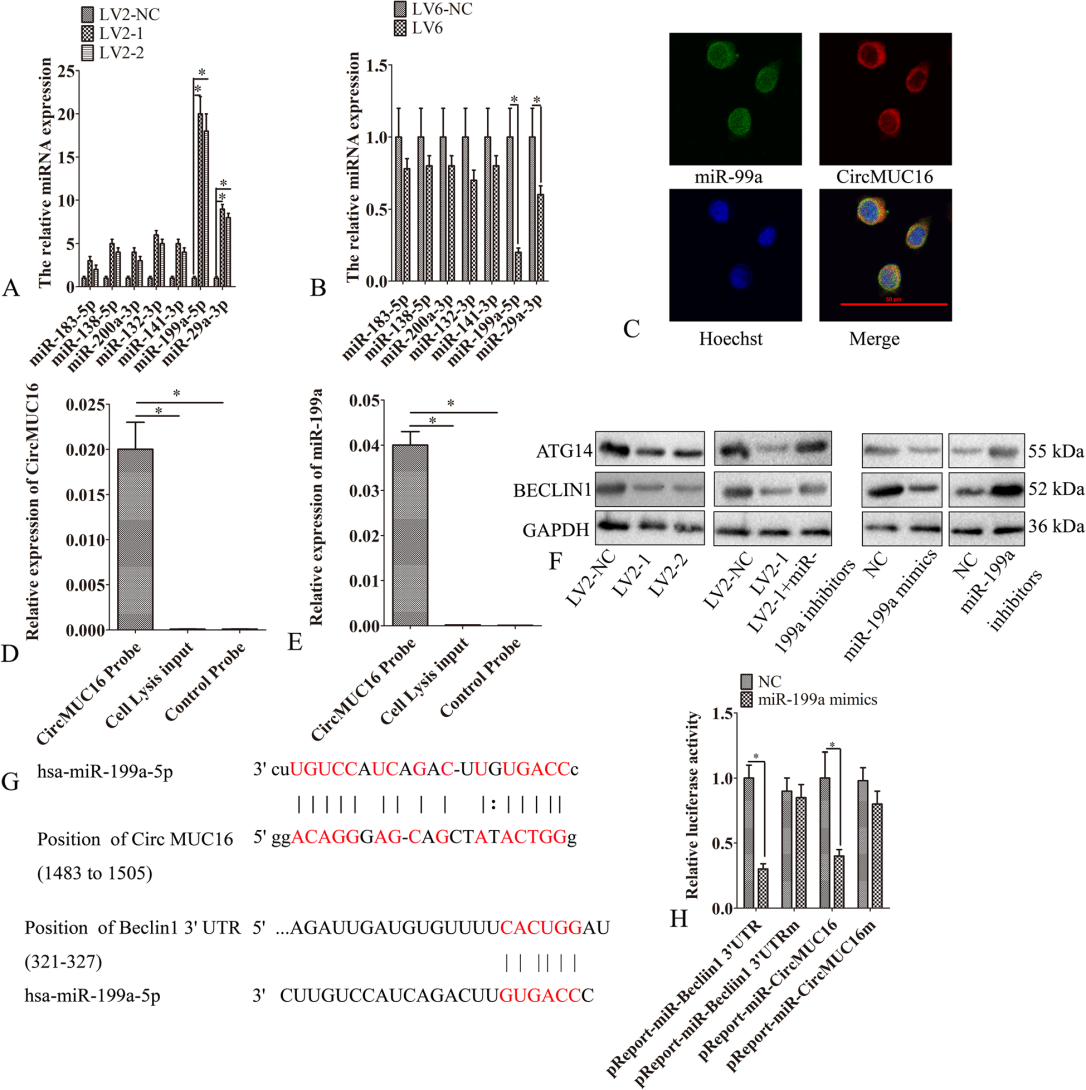
CircMUC16-miR-199a-5p-Beclin1 axis regulated autophagy. **a** The expression of miRNAs of SKOV3 cells was determined by qPCR. **b** The expression of miRNAs of A2780 cells was determined by qPCR. **c** The location of miR-199a and circMUC16 in SKOV3 cells was detected using FISH assay. **d** RNA pulled down assay was performed. Then, the expression of circMUC16 was detected by qPCR. **e** RNA pulled down assay was performed. Then, the expression of miR-199a was detected by qPCR. **f** The expression of Beclin1 and ATG14 of SKOV3 cells was determined using western blot. **f** SKOV3 cells were transfected with LV2-NC, LV2–1, LV2–2, LV2–1 + miR-199a inhibitors, miR-199a mimics, miR-199a mimic inhibitors. Then, the expression of Beclin1 and ATG14 were detected using western blot. **g** SKOV3 cells were co-transfected with miR-199a mimic or control RNA (NC) using luciferase reporter plasmids containing either wild-type (pMIR-CircMUC16) or mutant (pMIR-CircMUC16m). Luciferase expression was measured. Error bars represent the standard error. The symbols * and ** indicate *p* < 0.05 and 0.01, respectively

We predicted that circMUC16 might sponge multiple miRNAs (Miranda v3.3a). Hence, we detected the expression of miR-183, miR-138, miR-200a, miR-132, miR-141, miR-199a and miR-29a. Silencing circMUC16 significantly increased the miR-199a expression (Fig. 4a). Ectopic expression of circMUC16 significantly increased miR-199a expression (Fig. 4b). The FISH assay demonstrated that circMUC16 was mainly localized in the cytoplasm and overlapped with miR-199a (Fig. 4c). RNA pulldown assay showed that circMUC16 directly bound to miR-199a (Fig. 4d, e). Previous studies have demonstrated that Beclin1 and ATG14 were direct targets of miR-199a. Beclin1 was decreased expression in SKOV3 cells infected with LV2–1 or LV2–2 than LV2-NC. This regulation was abrogated when miR-199a-5p was silenced. We also demonstrated that miR-199a mimicked and inhibited the expression of ATG5 and Beclin1. The elevated expression of ATG5 and Beclin1 was observed after transfection with miR-199a inhibitors (Fig. 4f). The directly interaction between miR-199a, circMUC16 and Beclin1 was confirmed (Fig. 4g, h).

### CircMUC16 regulated RUNX1 via sponging miR-199a-5p

**Fig. 5:**
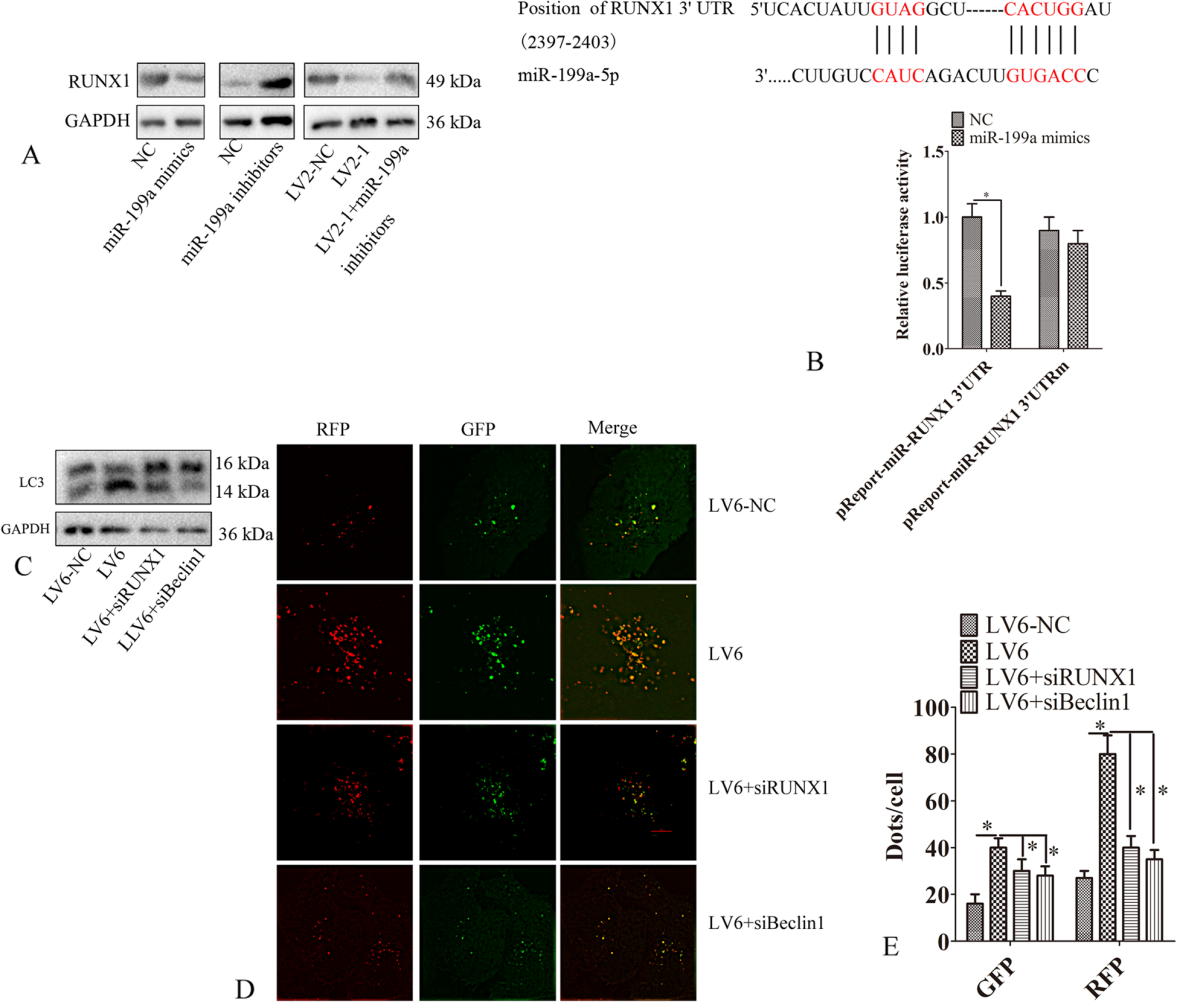
CircMUC16 regulated RUNX1 via sponging miR-199a-5p. **a** The expression of RUNX1 was determined by western blot. **b** SKOV3 cells were co-transfected with miR-199a mimic or control RNA (NC) using luciferase reporter plasmids containing either wild-type (pMIR-RUNX1 3’UTR) or mutant (pMIR-RUNX1 3’UTRm). Luciferase expression was measured. **c** A2780 cells were transfected with LV6, LV6-NC, LV6 + RUNX1 siRNA, or LV6 + Beclin1 siRNA. After 48 h, the expression of LC3-II was determined by western blot. **d**, **e** A2780 cells were transfected with LV6, LV6-NC, LV6 + RUNX1 siRNA, or LV6 + Beclin1 siRNA. Then, mRFP-GFP-LC3 distributions were determined by confocal microscopy. Error bars represent the standard error. The symbols * and ** indicate *p* < 0.05 and 0.01, respectively. Scale bar: 5 μm

Bioinformatics (Targetscan) predictions suggested that RUNX1 is a potential target for miR-199a-5p. Silencing miR-199a-5p increased the expression of RUNX1. However, ectopic expression of miR-199a-5p inhibited the expression of RUNX1 (Fig. 5a, b). RUNX1 was decreased expression in SKOV3 cells after silencing circMUC16. This regulation was abrogated after miR-199a-5p was knockdown (Fig. 5a). We observed that miR-199a-5p mimics significantly suppressed luciferase activity when RUNX1 3’UTR was inserted downstream of the luciferase cDNA (pMIR-RUNX1 3’UTR) (Fig. 5b). LC3-II was increased expression in A2780 cells infected with LV6. However, LC3-II expression was partly reduced when RUNX1 or Beclin1 was silenced (Fig. 5c). Moreover, circMUC16 promoted autophagy flux of A2780 cells. This effect was partially rescued via silencing RUNX1 or Beclin1 (Fig. 5d, e). These results indicated that circMUC16 regulated RUNX1 or Beclin1 via sponging miR-199a-5p.

### RUNX1 promoted the expression of circMUC16

**Fig. 6:**
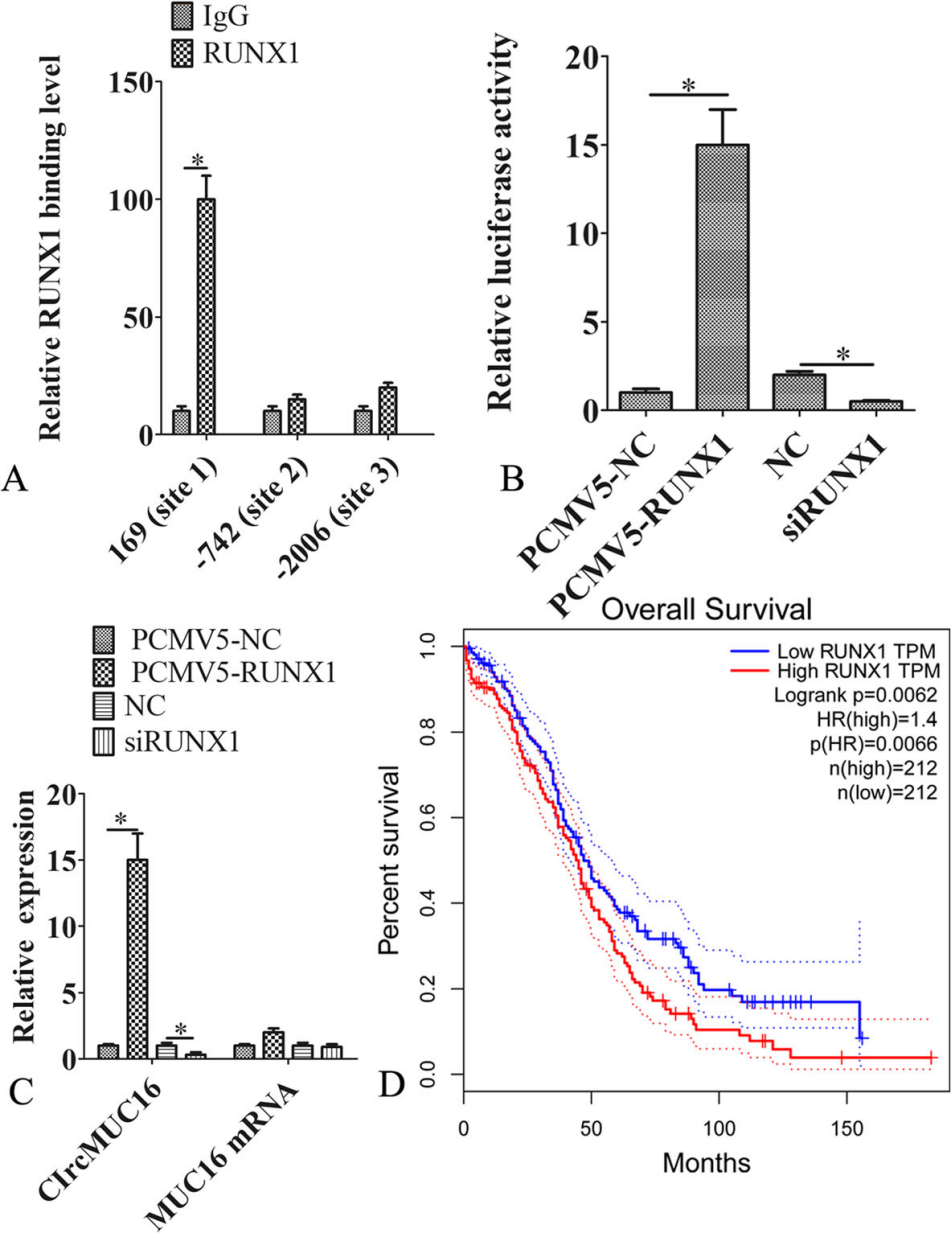
RUNX1 promoted the expression of circMUC16. **a** The putative binding sites between circMUC16 and RUNX1. **b** SKOV3 cells co-transfected with MUC16 reporter gene plasmid and NC (negative control), RUNX1 siRNA, PCMV5-RUNX1 or PCMV5-NC (empty vector). After 48 h, luciferase activity was measured. **c** SKOV3 cells transfected with NC (negative control), RUNX1 siRNA, PCMV5-RUNX1 or PCMV5-NC (empty vector). After 48 h, the linear mRNAs of MUC16 and circMUC16 were detected by qPCR. **d** Overall survival was analyzed in ovarian cancer patients based on TCGA data. All experiments were repeated three times. Error bars represent the standard error. The symbols * and ** indicate p < 0.05 and 0.01, respectively

A previous research found that circRNAs expression was regulated by transcription factors [17]. Bioinformatic analyses showed three potential RUNX1 binding sites (169, − 742 and − 2006) in the MUC16 gene. Based on chromatin immunoprecipitation (ChIP) assay, we confirmed that RUNX1 binds to the promoter region of MUC16 (Fig. 6a). The dual-luciferase reporter system was used to investigated the effect of RUNX1 on the activity of the MUC16 promoter. The data showed that RUNX1 overexpression increased MUC16 promoter activity, while RUNX1 knockdown decreased MUC16 promoter activity (Fig. 6b). Silencing RUNX1 inhibited the expression of circMUC16. However, ectopic expression of RUNX1 elevated the expression of circMUC16. In contrast, there was no significant change in the linear mRNA of MUC16 (Fig. 6c). We analyzed the overall survival (OS) of the ovarian cancer patients derived from TCGA online tool (http://gepia.cancer-pku.cn/). We found that the low expression of RUNX1 mRNA was significantly related to OS of the ovarian cancer patients (Fig. 6d).

### CircMUC16 directly binds to ATG13 protein

**Fig. 7:**
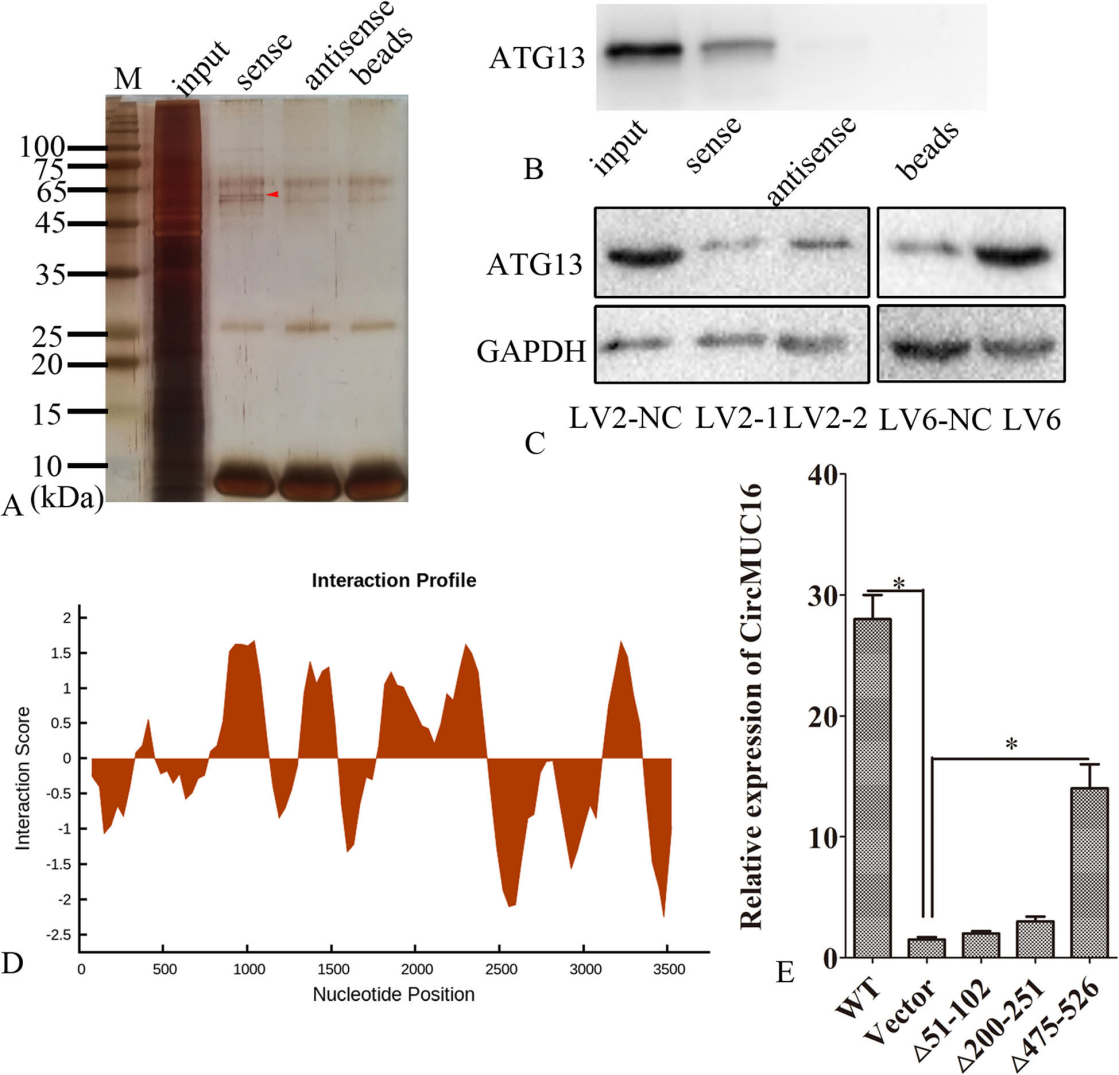
CircMUC16 directly bound to ATG13 protein. **a** The circMUC16 RNA pulldown was performed. Then, silver stains was obtained. **b** The circMUC16 specific binding protein gels was identified by RNA pulldown. **c** The expression of ATG13 protein was determined by western blot. **d** The potential interaction sites between circMUC16 and ATG13 protein were predicted using catRAPID. **e** Segments of ATG13 DNA were cloned into a PCMV5 plasmid. SKOV3 cells were transfected with PCMV5 vector, PCMV5-ATG13, PCMV5-△51–102, PCMV5-△200–251 or PCMV5-△475–526. After 48 h, the expression of circMUC16 was determined. Error bars represent the standard error. The symbols * and ** indicate p < 0.05 and 0.01, respectively

To further explore the mechanism of circMUC16-mediated regulation of autophagy, we performed RNA pulldown assay. The result of silver stain showed some differential bands (Fig. 7a). Using MALDI-TOF-MS, ATG13 protein was found. ATG13 directly bound to circMUC16 in the RNA pulldown assay (Fig. 7b). Silencing circMUC16 inhibited the expression of ATG13, while ectopic expression of circMUC16 promoted the expression of ATG13 (Fig. 7c). Based on catRAPID, we predicted that some region of ATG13 could bind to circMUC16 (Fig. 7d), and subsequently found that the △475–526 region of ATG13 directly bound to circMUC16 (Fig. 7e).

### CircMUC16 mediated metastasis of SKOV3 cells in vivo

**Fig. 8:**
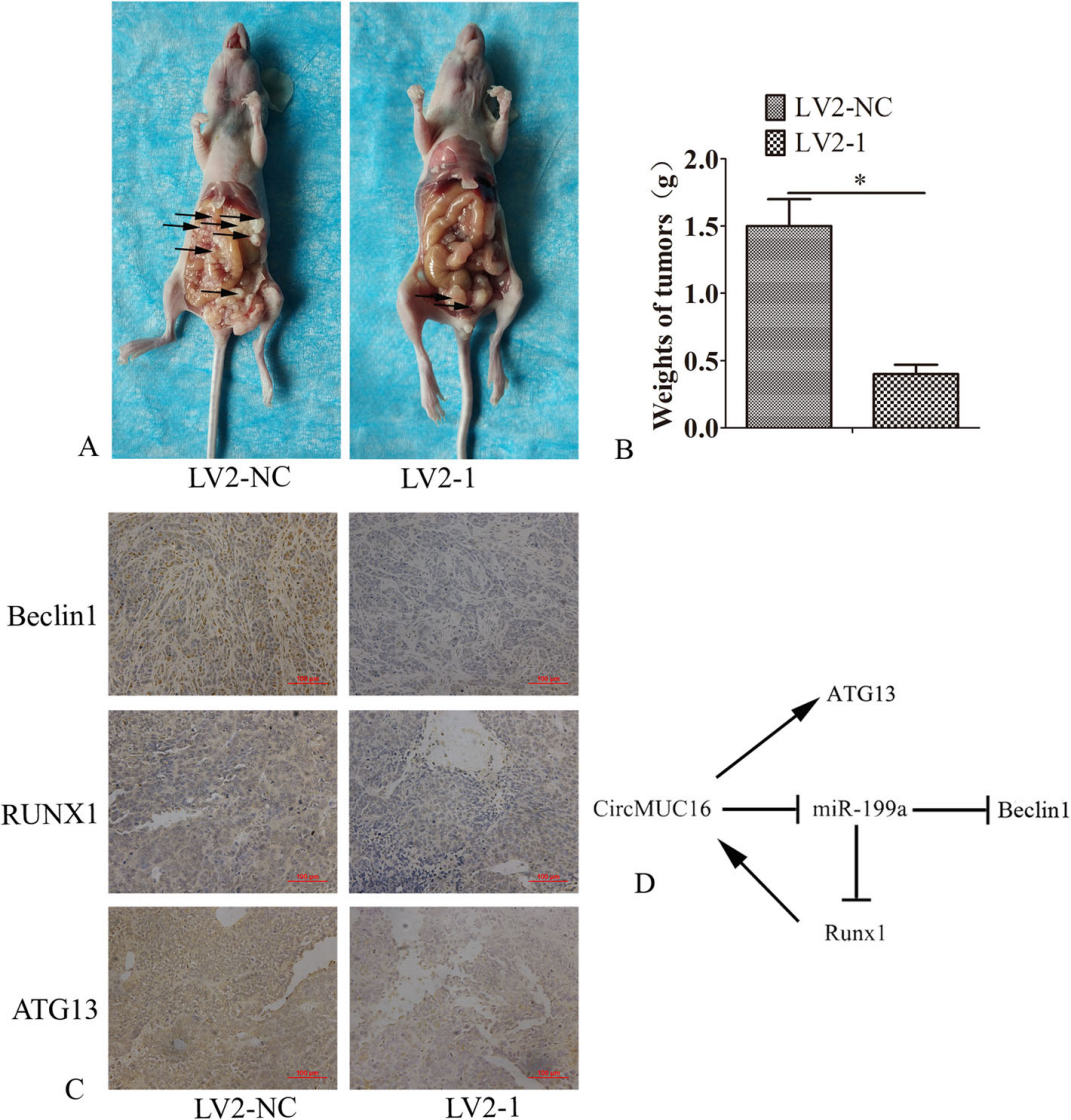
CircMUC16 mediated metastasis of EOC cells in vivo. **a** Silencing circMUC16 suppressed pelvic peritoneal invasion. The arrow showed the tumor in the abdominal cavity. **b** Mean tumor weight was calculated. **c** Immunohistochemical analysis of Beclin1, RUNX1 and ATG13 proteins. **d** A graphic abstract depicting the circMUC16 pathway (CircMUC16, RUNX1, Beclin1, ATG13 and miR-199a). All experiments were repeated three times. Error bars represent the standard error. The symbols * and ** indicate p < 0.05 and 0.01, respectively. Scale bar: 100 μm

The average weight of the lesions were reduced after CircMUC16 was knockdown (*p* < 0.05) (Fig. 8a, b). Immunohistochemistry results suggested that the expression of Beclin1, RUNX1 and ATG13 from the LV2–1 infected group was decreased as compared to the LV2-NC infected group (p < 0.05) (Fig. 8c). These results indicated that knockdown of circMUC16 suppressed the expression of Beclin1, RUNX1 and ATG13 in vivo. Based on the above results, we speculated that CircMUC16 promoted autophagy of epithelial ovarian cancer via Beclin1, RUNX1 and ATG13(Fig. 8d).

## Discussion

This study showed that circMUC16 was increased expression in EOC samples than healthy ovarian tissues. The expression of circMUC16 was related to tumor grade and stage. Furthermore, circMUC16 regulated Beclin1 or RUNX1 via sponging miR-199a-5p. In turn, RUNX1 promoted the transcription of circMUC16. Therefore, circMUC16 was a potential therapeutic target and diagnostic marker.

CircRNAs have been regarded as markers of many cancer types. In progressive laryngeal cancer, hsa_circRNA_104912 had increased expression and was a novel biomarker [18]. The expression of hsa_circ_0000190 was related to tumor progression, TNM stage and CA19–9 levels [19]. In this research, we found that circMUC16 was increased expression in EOC samples and sera from patients with EOC. The expression of circMUC16 correlated with tumor grade and stage. Therefore, circMUC16 was a potential biomarker for EOC.

CircRNAs typically regulate the target genes by sponging miRNAs. cir-ITCH enhanced ITCH expression via sponging miR-7 and miR-214, and then suppressed Wnt/β-catenin signaling pathway [20]. CircRNA-MYLK directly bound to and sponged miR-29a, and then relieved the suppression of target VEGFA [21]. This study showed that circMUC16 regulated Beclin1 and RUNX1 by sponging miR-199a-5p. miR-199a suppressed autophagy by GSK3β/mTOR complex signaling [22]. Moreover, miR-199a directly targeted Beclin1 to inhibited autophagy and reversed drug resistance induced by cisplatin of osteosarcoma cells [23]. Our results also demonstrated that miR-199a targetly regulated Beclin1, which was consistent with the previous study. We also verified that circMUC16-mediated autophagy accelerated the cellular proliferation and invasion. Therefore, circMUC16 acted as a cancer-promoting gene.

Silencing RUNX1 inhibited cellar proliferation and promoted of apoptosis [24]. miR-141 can retard cell proliferation and migration. miR-141 also promoted apoptosis of prostatic cancer. Over-expression of RUNX1 reversed this phenotype. RUNX1 was the direct target of miR-141. In this research, we observed that circMUC16 elevated the expression of RUNX1 via sponging miR-199a-5p. Knockdown of RUNX1 partly retarded the autophagy induced by circMUC16. We also demonstrated that RUNX1 facilitated the expression of circMUC16 by promoting transcription. Therefore, circMUC16-miR-199a-5p-RUNX1 feedback loop boosted autophagy of EOC.

In summary, to the best of our knowledge, this is the first report on circMUC16 being abnormally expressed in EOC. CircMUC16 was correlated with the tumor stage and grade. CircMUC16-mediated autophagy accelerated the malignant behavior of EOC. Hence, circMUC16 was a potential target for the diagnosis and treatment of ovarian cancer.

## Data Availability

All data generated or analyzed during this study are included in this published article.

## Abbreviations

circRNA: circular RNA
EOC: epithelial ovarian cancer
FISH: fluorescence in situ hybridization
